# Neonatal Tactile Stimulation Downregulates Dendritic Spines in Layer V Pyramidal Neurons of the WAG/Rij Rat Somatosensory Cortex

**DOI:** 10.1155/2022/7251460

**Published:** 2022-04-12

**Authors:** Gul Ilbay, Aymen Balıkcı, Sibel Köktürk, Melda Yardımoglu Yılmaz, Nurbay Ates, Canan Baydemır, Sibel Balcı

**Affiliations:** ^1^Department of Physiology, Kocaeli University, School of Medicine, Kocaeli, Turkey; ^2^Department of Histology and Embryology, Istanbul University, Istanbul Medical Faculty, Istanbul, Turkey; ^3^Department of Histology and Embryology, Kocaeli University, School of Medicine, Kocaeli, Turkey; ^4^Department of Biostatistics and Medical Informatics, Kocaeli University, School of Medicine, Kocaeli, Turkey

## Abstract

**Objective:**

The aim of our study is to examine the effects of neonatal tactile stimulations on the brain structures that previously defined as the focus of epilepsy in the Wistar-Albino-Glaxo from Rijswijk (WAG/Rij) rat brain with genetic absence epilepsy.

**Methods:**

In the present research, morphology and density of dendritic spines were analyzed in layer V pyramidal neurons of the somatosensory cortex (SoCx) of WAG/Rij rats (nonstimulated control, tactile-stimulated, and maternal separated rats) and healthy Wistar (nonepileptic) rats. To achieve this, a Golgi-Cox method was used.

**Results:**

Dendritic spine number in layer V of the SoCx has been detected significantly higher in adult WAG/Rij rats at postnatal day 150 in comparison to nonepileptic adult control Wistar rats (*p* < 0.001). Moreover, quantitative analyses of dendrite structure in adult WAG/Rij rats showed a decrease in dendrite spine density of pyramidal neurons of SoCx which occurred in early neonatal exposure to maternal separation (MS) and tactile stimulation (TS) (*p* < 0.001).

**Conclusions:**

Our findings provide the first evidence that tactile stimulations during the early postnatal period have a long-term impact on dendrite structure in WAG/Rij rat's brain and demonstrate that neonatal tactile stimulation can regulate dendritic spines in layer V in pyramidal neurons of SoCx in epileptic brains.

## 1. Introduction

The Wistar-Albino-Glaxo from Rijswijk (WAG/Rij) rat is an extensively utilized genetic model for generalized absence epilepsy with comorbid depression, in which cognitive impairment and poor maternal behavior have also been recently reported [1–3]. Additionally, they have been considered as an absence epileptogenesis animal model [1].

2–3-month-old WAG/Rij rats begin to manifest the spontaneously occurring 7–10 Hz spike-wave discharges (SWDs) in the cortical electroencephalogram (EEG) alongside decreased consciousness and immobility as in human absence epilepsy. All rats of this strain, at six months of age, show about 16-20 SWDs an hour [4]. WAG/Rij rats of the same age show depression-like behaviour symptoms, which tend to aggravate alongside with SWD increase [5].

It is broadly accepted that SWDs occur in an interconnected intact cortico-thalamo-cortical network. This network that comprises of the cerebral cortex, the thalamic relay nuclei, the intralaminar thalamic nuclei, and the reticular thalamic nucleus has a role in the appearance of synchronized and generalized SWDs in absence epilepsy [6]. SWD generation and occurrence are not completely understood. However, it appears that SWDs in genetic absence models have a cortical focal origin in the deep layers (layer V-VI) of the perioral region of the SoCx [1, 7].

Seizure activity begins in deep-layer pyramidal neurons, then spreads to upper-layer pyramidal cells (I and III), which propagate epileptic activity to ipsilateral cortical areas.

SoCx and other cortical regions transfer the ictal discharges to the sensory (ventro-posteromedial; posterior medial) and nonspecific thalamic (ventral medial) nuclei. The thalamus then acts as a resonator in thalamo-cortico-thalamic loops to maintain SWD. [1, 7, 8]. In addition, recent data suggest that impaired cortico-striatal excitatory transmission contributes to absence seizures. Notably, cortical layer V pyramidal neurons, which are a major source of excitatory inputs to the striatum, are thought to be involved in SWD generation [9].

Manipulations in maternal environment are well-known to affect epileptic activity in adult WAG/Rij rats that are genetically predisposed to absence epilepsy. Sitnikova (2011) demonstrated that whisker trimming during early life in WAG/Rij rats resulted in seizure activity increase in adulthood [10]. WAG/Rij rat pups that were handled for 15 mins during postnatal days 1-22 demonstrated a decrease in SWD at adult ages. During the same postnatal period, the reduction in SWD number was observed in pups that were exposed to maternal deprivation for 180 minutes [11]. It should be mentioned that these two procedures commonly increase maternal care.

It has recently been shown that maternal care decreases the number and mean duration of SWDs and delays the appearance and progression of absence epilepsy. Increased early maternal care not only exerts antiepileptogenic effects but also counteracts the development of comorbid depression in adult WAG/Rij rats [12]. A previous study by our group has shown that tactile stimulation (TS) during postnatal period in WAG/Rij rats genetically predisposed to epilepsy reduced epileptic activity and comorbid depression in adulthood [13].

The outcomes of these studies show that an increase in tactile stimulations in initial development periods forms absence seizure-modifying effects in WAG/Rij rats. Application of TS mimics maternal licking and grooming behavior in rats, which is a sensory stimulation method to the skin [14]. Studies show that TS therapy stimulates maturation in rat pups and in human infants [15]. Evidence has shown that TS during initial periods of development enables to reorganize dendritic organization in various brain regions and induces behavioural benefits in adult age [16]. Given during early developmental periods, TS improves anxiety-like behaviors and prevents preference to addictive drugs and depression-like behaviors [14]. When given in adult rats, however, TS shows beneficial influence on the brain function, preventing cortical lesion and increasing neurotrophin and dendritic length [17]. In addition, Schridde et al. showed that neonatal handling and maternal deprivation significantly decreased absence seizure and led to an increase of HCN hyperpolarization-activated cation channel 1 (HCN1) and hyperpolarization-activated cation current (*I*_h_) in the layer V pyramidal neurons where SWDs are thought to originate [11]. In the current literature, SWDs in WAG/Rij rats have been linked to a decrease in HCN/*I*_h_ in the layer V of the SoCx [11, 12].

Based on this background, we hypothesized that the effects of neonatal tactile stimulations on absence seizure might be associated with changes in neuronal organization. Therefore, in this study, we examined the effects of the neonatal TS on dendritic morphology in layer 5 pyramidal neurons of SoCx in genetically predisposed WAG/Rij rat's brain in adulthood. To achieve this, a Golgi-Cox method was used. SoCx layer V was chosen for analysis because in WAG/Rij rats, it has been shown that spontaneous SWDs have their onset in this region and for layer V being sensitive for early environmental manipulations [11].

## 2. Material and Methods

### 2.1. Animals and Study Design

Each pregnant female Wistar and WAG/Rij rat from the breeding facility of University of Kocaeli was housed in a Plexiglas cage and had free access to food and water under controlled temperature of 22-23°C and on a 12 : 12 h light : dark cycle.

The births were monitored, and at postnatal day one, male pups of litters were allocated at random to four groups: control Wistar (not touched) rats, control WAG/Rij (not touched) rats, maternally separated WAG/Rij rats, and tactile-stimulated WAG/Rij rats. For randomization, male WAG/Rij pups of six litters were placed together in a warm plastic box and then were returned to the lactating dams. Wistar control pups were collected from two different litters randomly. At postnatal day one, male rat pups were identified using the anogenital distance. It is longer in males than females, approximately 3.6 mm versus 1.8 mm [12]. Female WAG/Rij rats were not preferred for this study because hormonal fluctuations that occur during the estrous cycle can affect many physiological processes, including spinogenesis [18, 19].

From postnatal day 3 to day 21, TS was applied three times per day (9:00 AM, 1:00 PM, and 4:00 PM). At each session, mothers stayed in a different cage with food and water. A soft baby brush was used to brush each pup in the TS group during 15 minutes [20].

In maternal separation (MS), pups remained in a warm plastic box without their mother during 15 minutes three times per day (9:00 AM, 1:00 PM, and 4:00 PM) between P3 and P21. By the end of procedure, rat pups were placed back with their mothers. Maternal separation is a tactile deprivation procedure which subsequently causes increased maternal care. The control groups stayed in their cage without receiving any stimulation and were only handled during the home cage regular cleaning, two times a week. Following TS and MS on P21, pups were weaned and housed in groups. Body weight of rat pups was monitored each week as weight loss or decreased food intake were the study criteria for exclusion. All attempts were made to use the least number of animals in the experiments and minimize their suffering from experimental procedures. The experiment was approved by the Ethical Committee on Animal Experimentation of the University of Kocaeli, Turkey (KOÜ-HADYEK 6/5-2016).

### 2.2. Golgi-Cox Staining Procedure

**Figure 1:**
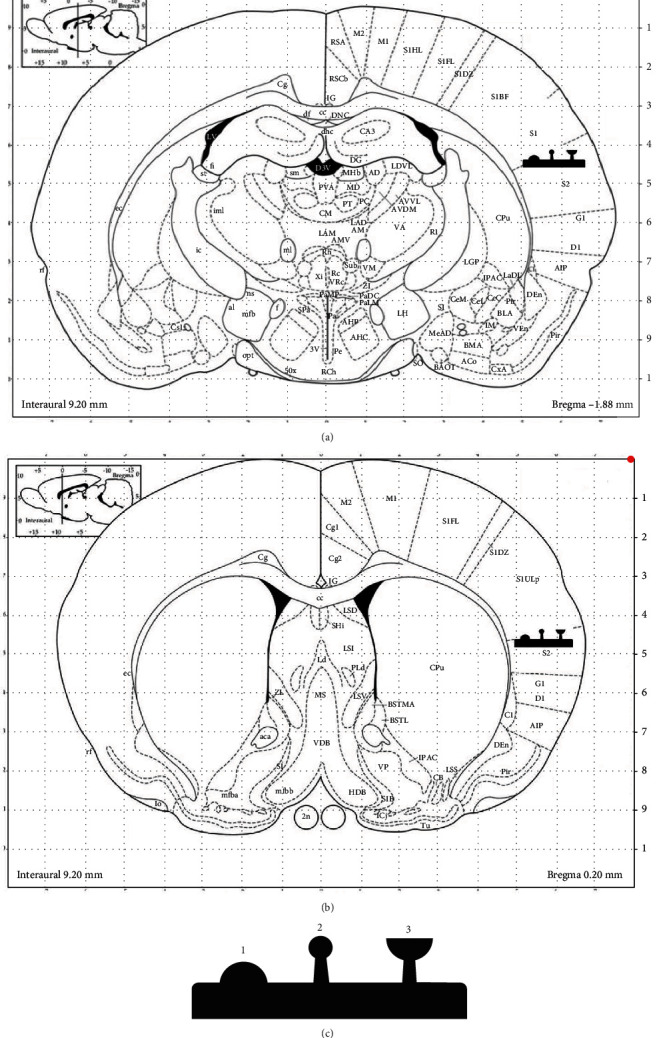
Investigated two areas in the somatosensory cortex are indicated by “
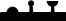
” icon. (a) The secondary somatosensory (S2) area (AP 0.0 ML 8.0). (b) The edge of the secondary somatosensory area and vibrissae (AP-2.0 ML 7.0) (Paxinos and Watson, 2007). (c) Dendritic spine morphology icon: Stubby (1), thin (2), and mushroom (3) spine subtypes.

At PND 150, a total of 24 adult rats (6 rats in each group) were deeply anesthetized with 100 mg/kg intraperitoneal injections of sodium pentobarbital and sacrificed by decapitation using a rodent guillotine. The corneal reflex was used to determine the deep level of anesthesia. After decapitation, the brains were removed. We used Hito Golgi-Cox OptimStain Kit (Hitobiotec Inc., Wilmington, DE, USA) for tissue preparation and staining method. The staining method was carried out according to the manufacturer's user manual. A series of 50-80 *μ*m coronal sections was obtained from the SoCx that contains the epileptic zone [21]. In quantification of dendritic spine density, coordinates were provided from Paxinos and Watson atlas (2007): for the first point to bregma AP 0.0 ML 8.0; for the second point from IL from bregma AP −2.0 ML 7.0. (Figures 1(a) and 1(b)) [22].

Three images that cover the whole thickness of every dendrite were provided by making use of a 100x oil immersion objective on a bright-field microscope (Leica DM2500). Fragments of individual randomly selected dendrites were quantified in order to measure spine density. The Golgi-Cox stained brains were randomly chosen the three distinct pyramidal neurons from each animal in the all groups for analysis of spine density. In the deep layer (V) pyramidal neurons of the SoCx was analyzed the randomly chosen 30 *μ*m length segment from the apical dendrite for spine morphological categorization and density (54 dendrites per group). Spine densities of the SoCx were analyzed by making use of the Image J software (National Institutes of Health, Bethesda, MD, USA). Quantification was carried out blindly and for each animal, the average spine density values (number of spines/*μ*m) were measured. Morphometric analysis was conducted for each spine, and measurements categorized spines into stubby, thin, and mushroom (Figure 1(c)) subtypes.

### 2.3. Statistical Analysis

Average values for each animal and experimental group were used for the statistical analyses. Numeric variables (the dendritic spine and the number of each spine type) were presented with median (interquartile range). Kolmogorov-Smirnov test was used to assess the assumption of normality. Since the normality assumption did not hold, Kruskal-Wallis test was used to compare the groups (Wistar control, WAG/Rij control, MS and TS) with respect to the dendritic spine and the number of each spine type. Dunn's test was used for the multiple comparisons. All statistical analyses were performed using IBM SPSS for Windows version 20.0 (IBM Corp., Armonk, NY, USA). A *p* value < 0.05 was considered statistically significant.

## 3. Results

**Figure 2:**
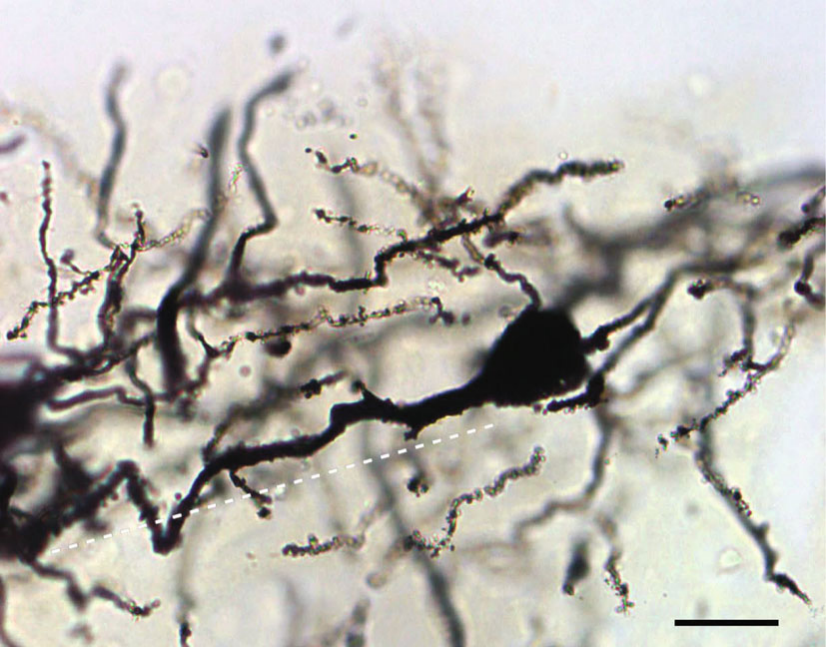
Photomicrograph demonstrating Golgi-Cox impregnated pyramidal neurons of the somatosensory cortex from control adult WAG/Rij rats. Apical dendritic segment is indicated by dashed white line. Scale bar, 20 *μ*m.

The dendritic shafts and spines of deep layer pyramidal neurons of the SoCx were distinctly observed by the Golgi-Cox staining procedure (Figure 2).

**Figure 3:**
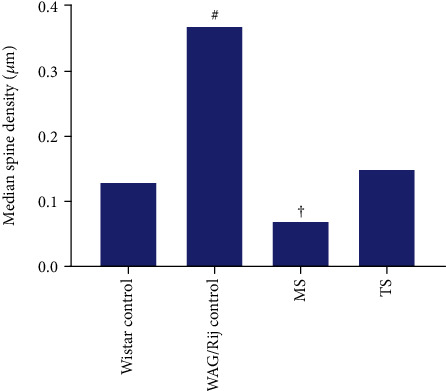
Median spine density in layer V pyramidal neurons of control Wistar (*n* = 6), control WAG/Rij (*n* = 6), maternally separated WAG/Rij (*n* = 6), and tactile-stimulated WAG/Rij rats (*n* = 6). ^#^*p* < 0.001, WAG/Rij control compared to other groups and ^†^*p* < 0.001, MS compared to other groups (Kruskal Wallis test).

The dendritic spine density of the SoCx pyramidal cells was different between WAG/Rij control and nonepileptic Wistar rats. The SoCx of WAG/Rij control displayed significantly higher median spine density compared with control Wistar rats (*p* < 0.001) (Figure 3) (Table). Repeated exposure to TS and MS early in life reduced dendritic spine density in layer V of SoCx in WAG/Rij rats (*p* < 0.001). Neonatal TS in WAG/Rij rats caused dendritic spine density of the SoCx layer V to downregulate to the levels of Wistar rats (*p* > 0.05). On the other hand, MS rats had lower spine density than Wistar control rats and tactile-stimulated rats (*p* < 0.001) (Figure 3) (Table).

**Figure 4:**
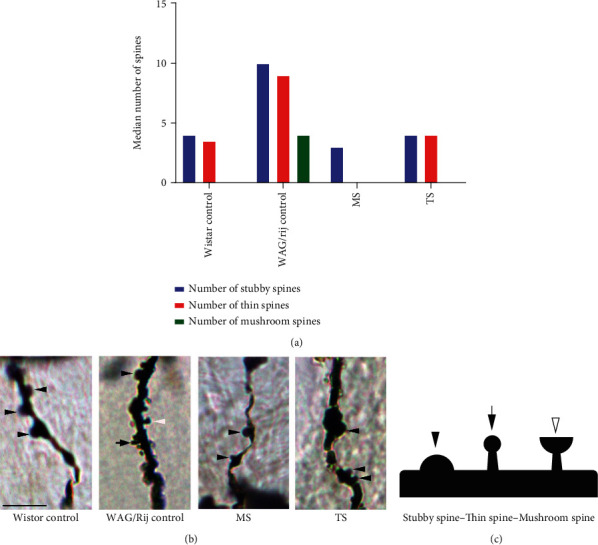
(a) Median number of spine subtypes in layer V pyramidal neurons of control Wistar, control WAG/Rij, maternally separated WAG/Rij, and tactile-stimulated WAG/Rij rats. (b) Photomicrographic (2000x) representation of different dendritic densities and dendritic spine types in four groups. The magnifications show examples of each dendritic spine type. (c) In the bottom, a schematic representation of each dendritic spine type is presented and matched with related arrows. Scale bar 5 *μ*m (b).

Stubby, thin and mushroom spines were the most common types found in deep layer (V) of the rat SoCx of control Wistar and control WAG/Rij rats. As shown in the table, there were marked differences in the number of mushroom type of dendritic spine between control Wistar and control WAG/Rij rats. Mushroom spines were higher in the layer V SoCx of control WAG/Rij rats compared to Wistar control rats (*p* < 0.001). In tactile-stimulated WAG/Rij rats, the number of mushroom spines were reduced to the level of Wistar control (*p* > 0.05). MS reduced number of stubby and thin subtypes when compared to other groups (*p* < 0.001), and also, number of mushroom spines of MS rats was significantly less than the WAG/Rij control rats (*p* < 0.001) (Figure 4) (Table 1).

## 4. Discussion

Our present results showed that, in deep layer V of SoCx, WAG/Rij rats had a higher density of dendritic spine when compared to healthy Wistar rats. Furthermore, our results showed for the first time that sensory experiences in early developmental period can cause permanent changes in dendritic spine density in WAG/Rij rats. A decrease occurred in the density of dendritic spines in the deep layer V of SoCx in adult WAG/Rij rats when subjected to TS and MS during neonatal period. However, TS regulated the spine density to the levels of nonepileptic control Wistar rats, while MS decreased spine densities compared to other groups, including control Wistar rats. There were also differences in the spine morphology amongst groups, especially the mushroom type spines were the most prominent in the WAG/Rij control group. Additionally, sensory experiences induced changes in spine morphology within the SoCx in all groups. In the layer V pyramidal cells, all spine types were decreased in the maternally separated animals while TS only reduced mushroom type spines.

The SoCx in rats that are genetically predisposed to absence epilepsy is regarded to trigger epileptic discharges. In general, it was concluded that the spike-wave discharges firstly appear at SoCx and then rapidly spreads to the remaining parts of the cortex and cortico-thalamic network. Seizure activity initially takes place in the deep-pyramidal cells, afterwards, in the superficial layer pyramidal neurons and spreads towards ipsilateral cortical areas [8].

The SoCx in WAG/Rij rats is characterized by synaptic hyperexcitability [10]. In absence seizures, electrophysiological intracellular recordings demonstrated that the pyramidal cells in the deep layers of SoCx show fast activation, hyperexcitability, and hypersynchronizing characteristics [8]. Karpova et al. (2005) demonstrated significant structural changes in dendritic and axonal arborization in pyramidal neurons SoCx of WAG/Rij rats. In WAG/Rij rats, it was found that there were longer dendrites and had less branching in pyramidal neurons of SoCx compared with nonepileptic control rats [23]. According to these data that were collected in the upper layers, it is suggested that epileptic rats might have atypically characterized neurons in the SWD generation site with greater arborizations and more synaptic connections between neurons. These features might facilitate the initiation and spreading of SWD [1].

In our experiment, different from previous study (Karpova et al., 2005), the neuronal organization of deep layer V of SoCx was investigated by Golgi-Cox staining in WAG/Rij rats with genetic epilepsy and in nonepileptic control rats with the same age. Similarly, we found quantitative differences in the density of dendritic spines between WAG/Rij rats and nonepileptic control rats in the deep layers V of SoCx. It is reasonable to consider that dendritic abnormalities may be the cause or the result of seizures.

Dendritic spines are thin protrusions that emerge from the surface of various neurons, specifically postsynaptic structures, which play mostly an excitatory role in synaptic communications [24, 25]. The morphology and density of dendritic spine are crucial for synaptic plasticity. Physiological and pathological conditions are reported to be associated with the spine morphology and density [25, 26]. Dendritic spine abnormalities are generally reported in the brain specimens of epileptic patients in hippocampal tissue of epilepsy patients with temporal lobe; decrease in dendritic spine density is frequently reported. However, alterations in dendritic length, shape, and branching patterns and focal increase in dendritic spines are less frequently reported in cortical and hippocampal tissues. In various animal models that involve acute seizures or chronic epilepsy, similar dendritic changes have been observed, which are primarily loss of dendritic spines and less frequently increase in dendritic spines [27].

Generally, the majority of excitatory synaptic inputs are received and integrated by dendritic spines in the central nervous system and therefore have influence on neuronal excitability. In certain kinds of epilepsy, hyperexcitable circuits and seizures might result from dendritic spine abnormalities. Therefore, it is safe to assume that high dendritic spine number and associated excitatory synaptic input disturb the balance between excitation and inhibition in the WAG/Rij rat brain, causing seizure. In addition, types of spine may have distinct functions, and alterations in the spine type ratio may cause a significant effect on neuronal excitability and function [28, 29]. Additional studies are needed to determine functional implications of these dendritic abnormalities in this genetic model.

In our study, all types of spines were reduced in epileptic rats exposed to MS. On the other hand, only the mushroom spines decreased in tactile stimulated WAG/Rij rats. It is known that mushroom spines generally have large heads with big spines and are characterized by the presence of large, very effective and strong synapses containing numerous glutamate receptors. Moreover, mushroom spines are more stable than other spines [30–32].

Therefore, our findings indicate that tactile stimulations modulate structural plasticity in the layer V of SoCx not only by decreasing spine numbers but also by changing spines types.

It is not surprising that tactile stimulations led to change spine density in the SoCx, where tactile sensation gets processed [21]. Various data confirm the beneficial effects of enriched environment on synaptic plasticity in different animal models [33, 34].

Studies showed that TS, which is an enriching positive experience that mimics maternal licking and grooming, has the potential to affect the neuroanatomic organization of the brain. Early life is a critical period for development of the central nervous system when plasticity levels are high and brain is extremely susceptible to environmental factors. Environmental stimuli during the early developmental period may influence the brain's functional maturation and its long-term integrity [14, 20, 35–37].

Richards et al. (2012) showed that TS early in life increased spine density, dendritic branching, and dendritic length in prefrontal cortex and amygdala of rats [14]. In another study, TS treatment, by increasing dendritic complexity, length, and synaptic contact in all cortical areas and amygdala, reversed neuroanatomical alterations caused by prenatal valproic acid exposure in rats [35]. However, Kolb and Gibb (2010) demonstrated that TS may differently alter synaptic organisation in different brain areas [36]. According to their study, TS treatment early in life decreases spine density and dendritic length in the parietal cortex. The same environmental exposure can alter spine density differently in accordance with manipulation age and manipulations early in life lead to decrease in spine density while those given in adulthood cause it to increase [38].

Interestingly, in SoCx, while MS decreased dendritic spine density, TS regulated spine density to the level of control Wistar rats. These results suggest that TS is more effective than increased compensatory licking and grooming behaviors (tactile stimulations) by the dams to the pups after the separation period [39].

It has been suggested that possible mechanisms that underlie the neural and behavioral effects of TS include endocrine function alterations, increased production of neurotrophic factors (insulin-like growth factor, brain-derived neurotrophic factor, and fibroblast growth factor-2), and altered gene methylation [14].

Further studies are required to clarify mechanisms underlying the seen effects of TS on the morphology and density of dendritic spines in the deep layer V of SoCx in epileptic rat's brain. It will also be interesting to investigate whether any structural differences between the apical and basilar spines occur as a result of tactile stimulation.

## 5. Conclusions

Our study provides additional evidence for the structural changes of SoCx pyramidal neurons in genetically absence epileptic rat's brain. We found that the dendritic spine density in the WAG/Rij rat strain was higher than Wistar rats (nonepileptic). Tactile stimulations downregulated dendritic spines and changed the morphology of the spines in WAG/Rij rat's brain. These results indicate that tactile stimulations may exert an important effect on the morphology and density of dendritic spines in the deep layer V of SoCx and may affect epileptic focus through the neuronal plasticity.

## Figures and Tables

**Table 1:** Comparison of the groups with respect to the dendritic spine and the number of each spine type.

Groups	Dendritic spine density	Number of stubby spines	Number of thin spines	Number of mushroom spines
Wistar control	0.13 (0.13-0.17)	4 (3-5)	3.5 (0-5)	0 (0-0)
WAG/Rij control	0.37 (0.33-0.40)	10 (0-12)	9 (0-11.25)	4 (0-11)
MS	0.07 (0.06-0.10)	3 (2-3)	0 (0-1.25)	0 (0-0.25)
TS	0.15 (0.13-0.17)	4 (3.75-5)	4 (2.25-5)	0 (0-0)
**p** **value**	**p** < 0.001^**a,b,c,d,e**^	**p** < 0.001^**a,b,c**^	**p** < 0.001^**a,b,c**^	**p** < 0.001^**c,d,e**^

Data were presented with median (interquartile range). Boldface *p* values indicated the statistically significant differences. ^a^MS vs. Wistar control; ^b^MS vs. TS; ^c^MS vs. WAG/Rij control; ^d^Wistar control vs. WAG/Rij control; ^e^TS vs. WAG/Rij control.

## Data Availability

The data used to support the findings of this study are available from the corresponding author upon request.
